# Antibody-Mediated Activation of FGFR1 Induces FGF23 Production and Hypophosphatemia

**DOI:** 10.1371/journal.pone.0057322

**Published:** 2013-02-22

**Authors:** Ai-Luen Wu, Bo Feng, Mark Z. Chen, Ganesh Kolumam, Jose Zavala-Solorio, Shelby K. Wyatt, Vineela D. Gandham, Richard A. D. Carano, Junichiro Sonoda

**Affiliations:** 1 Department of Molecular Biology, Genentech, Inc., South San Francisco, California, United States of America; 2 Biomedical Imaging, Genentech, Inc., South San Francisco, California, United States of America; Nihon University School of Medicine, Japan

## Abstract

The phosphaturic hormone Fibroblast Growth Factor 23 (FGF23) controls phosphate homeostasis by regulating renal expression of sodium-dependent phosphate co-transporters and cytochrome P450 enzymes involved in vitamin D catabolism. Multiple FGF Receptors (FGFRs) can act as receptors for FGF23 when bound by the co-receptor Klotho expressed in the renal tubular epithelium. FGFRs also regulate skeletal FGF23 secretion; ectopic FGFR activation is implicated in genetic conditions associated with FGF23 overproduction and hypophosphatemia. The identity of FGFRs that mediate the activity of FGF23 or that regulate skeletal FGF23 secretion remains ill defined. Here we report that pharmacological activation of FGFR1 with monoclonal anti-FGFR1 antibodies (R1MAb) in adult mice is sufficient to cause an elevation in serum FGF23 and mild hypophosphatemia. In cultured rat calvariae osteoblasts, R1MAb induces FGF23 mRNA expression and FGF23 protein secretion into the culture medium. In a cultured kidney epithelial cell line, R1MAb acts as a functional FGF23 mimetic and activates the FGF23 program. siRNA-mediated *Fgfr1* knockdown induced the opposite effects. Taken together, our work reveals the central role of FGFR1 in the regulation of FGF23 production and signal transduction, and has implications in the pathogenesis of FGF23-related hypophosphatemic disorders.

## Introduction

Inorganic phosphate (phosphorus) plays a crucial role in many biological processes including bone mineralization, vascular function, and cellular activity; therefore, its level in the body must be tightly regulated. Fibroblast Growth Factor 23 (FGF23) is an endocrine member of the FGF superfamily produced by osteocytes in the bone [1]–[3]. It acts as an important determinant of phosphate homeostasis by controlling renal phosphate transport as well as vitamin D catabolism. These activities are mediated, at least in part, by transcriptional regulation of genes encoding Na-dependent phosphate co-transporters, NPT2a and NPT2c, as well as cytochrome P450 enzymes, CYP24a1 and CYP27b1, that are respectively involved in the production and the catabolism of active vitamin D (1, 25(OH)_2_D_3_, calcitriol) in the kidney [4]–[6]. Genetic disorders with altered circulating levels of phosphate are often associated with dysregulation of the FGF23 pathway. For example, overproduction of FGF23 in osteoblastomas or stabilizing mutations in FGF23 protein is sufficient to cause hypophosphatemia, leading to osteomalacia or hypophosphatemic rickets in humans [5], [7]. In addition, inactivating mutations in genes such as *dentin matrix acidic phosphoprotein 1* (*Dmp1*) [8], *Phosphate regulating endopeptidase homolog, X-linked* (*Phex*) [9], or *Ecto-nucleotide pyrophosphatase/phosphodiesterase 1* (*Enpp1*) [10], [11], have been shown to increase skeletal secretion of FGF23, resulting in hypophosphatemic rickets.

The biological effects of FGF23 are mediated by receptor complexes formed by the membrane bound co-receptor Klotho protein and one of the FGFR isoforms [12], [13]. Klotho protein is highly expressed in the kidney and is absolutely essential for FGF23 signaling [14]–[16]. Although it is certain that FGFRs are also important for mediating the activity of FGF23, the relative contribution of each FGFR isoform remains unclear. Of the 7 primary isoforms of FGFR encoded by mammalian species (1b, 2b, 3b, 1c, 2c, 3c, and 4), FGFR1c, 2c, 3c and 4 can interact with Klotho to form functional receptor complexes for FGF23 [13]. Of these, FGFR1, 3, and 4, but not FGFR2, are detected in the renal proximal tubule where FGF23 is thought to regulate phosphate reabsorption [17]. Genetic studies suggest that each of these three FGFRs plays overlapping role in mediating the activities of FGF23. In one study, WT, *Fgfr3* KO, and *Fgfr4* KO mice responded to recombinant FGF23 injection by reducing serum phosphate levels as well as the expression of NPT1a and NPT2c proteins in renal cortex; however, conditional *Fgfr1* KO mice, deficient for FGFR1 in *Pax3* expressing metanephric mesenchyme including the renal proximal tubule, did not [17]. This suggests the predominant role of FGFR1 in mediating FGF23 effect in the kidney. In a subsequent study, however, FGF23 did not reduce serum phosphorus levels in double *Fgfr3/Fgfr4* KO mice [18], suggesting that FGFR3 and FGFR4 play redundant roles in phosphate regulation, and perhaps that FGFR1 activation is not sufficient for FGF23 to induce hypophosphatemia.

In addition to mediating the activity of FGF23, FGFRs are likely involved in regulating FGF23 production in bones. Gene expression analysis of *Phex*-deficient bone or differentiated bone marrow stromal cells lacking functional Phex or Dmp1 revealed an ectopic activation of FGFR pathway [19], [20]. Pharmacological inhibition by pan-FGFR inhibitors in these cells suppressed expression of FGF23. In addition, FGF1 and FGF2 increased FGF23 promoter activity in a reporter assay in osteoblastic cells [19], [20]. Although it is not clear which of the FGFRs mediate the effect of FGFR inhibitor or FGF ligand in the bone, an involvement of FGFR1 has been suggested. An increase in serum FGF23 has been reported for one case of a rare genetic hypophophatemic disorder called osteoglophonic dysplasia (OGD) caused by a dominant activating FGFR1 mutation [21]. However it is important to note that OGD caused by an *FGFR1* mutation is so rare that it is not clear whether the increase in FGF23 is a general feature of FGFR1 activation.

Previously we used phage-display technology and identified two monoclonal anti-FGFR1 antibodies (R1MAb1 and R1MAb2) that bind and activate both b and c isoforms of FGFR1 *in vitro* and *in vivo* [22]. Both R1MAbs activate FGFR1 in adipose tissues and induce sustained insulin sensitizing effects when injected into adult mice. In addition, we observed a mild, but significant reduction in serum phosphate levels in mice treated with R1MAb1 [22]. Here, we investigate the mechanism of phosphate regulation by R1MAbs and demonstrate that R1MAb perturbs phosphate regulation in two cell types; by regulating the production of FGF23 in bone cells, and by activating the FGF23 pathway to alter the expression of NPTs and CYP enzymes in renal epithelial cells. These findings add new insights into the biology of FGF23 pathway and pathogenesis of hypophosphatemic disorders. In addition, our findings have implications in the development of pharmacological agents that target FGFR1.

## Results

### R1MAb activates FGF23 pathway *in vivo*

**Figure 1:**
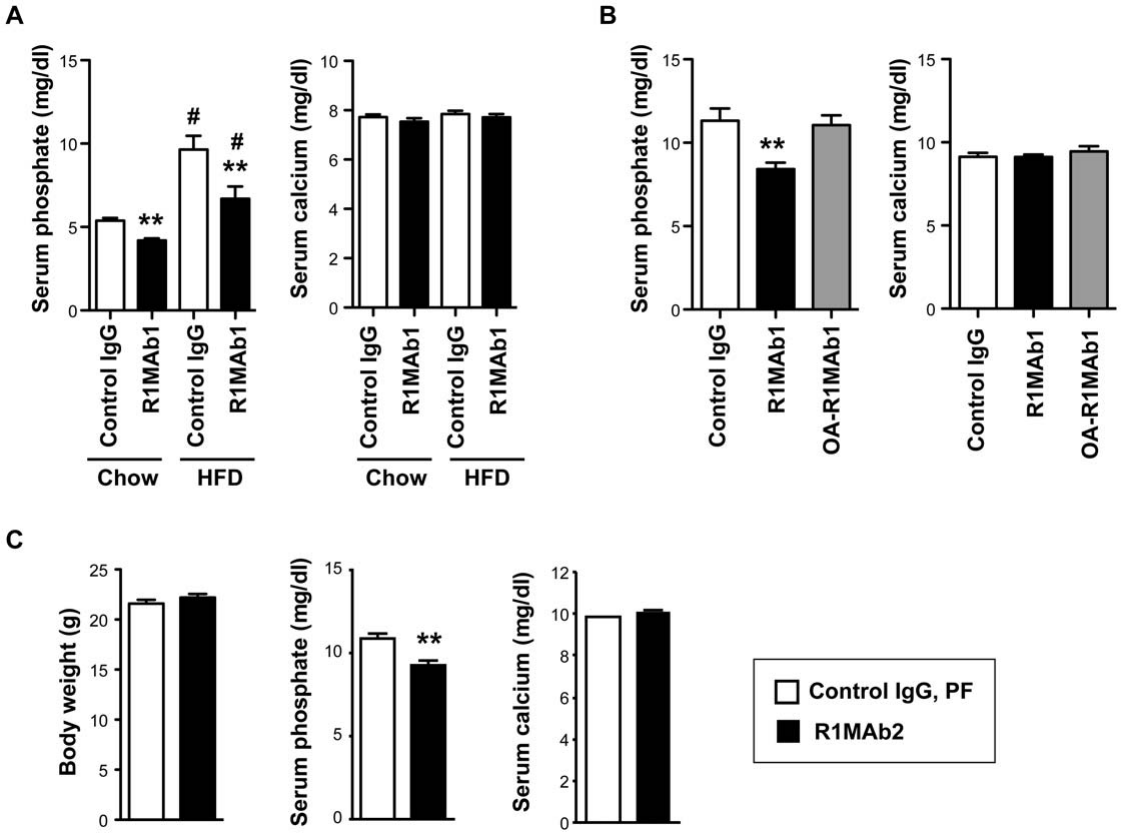
R1MAbs induce hypophosphatemia. (**A**) Serum phosphate and calcium levels in male C57BL/6 mice intraperioneally injected with R1MAb1 or isotype control (Control IgG) at 3 mg/kg. HFD-fed mice were on the diet for 15 weeks at the end of the study. Serum phosphate and calcium levels were determined at 7 days post injection. N = 8 mice/group. (**B**) Serum phosphate and calcium levels in female *db/db* mice intraperioneally injected with R1MAb1, OA-R1MAb1 or isotype control (Control IgG) at 3 mg/kg. Serum phosphate and calcium levels were determined at 7 days post injection. N = 5–7 mice/group. (**C**) Body weight, and serum phosphate and calcium levels in male C57BL/6 mice intraperioneally injected with R1MAb2 or isotype control (Control IgG) at 1 mg/kg. Control mice were subjected to pair feeding to adjust body weight. Body weight, serum phosphate and calcium levels were determined at 48 hour post injection. N = 8 mice/group. (A–C) * p<0.01, ** p<0.005 (versus control IgG). #<0.005 versus chow-fed group with the same antibody treatment.

Previously, we reported a mild reduction in serum phosphate levels in lean C57BL/6 and diabetic *db/db* mice treated with an agonistic anti-FGFR1 antibody, R1MAb1, at 0.5 mg/kg [22]. The activity of R1MAb1 to reduce serum phosphate levels was re-evaluated by injecting R1MAb1, or isotype control antibody, at 3 mg/kg into either lean C57BL/6 or high fat diet (HFD) fed C57BL/6 mice. In both cases, R1MAb1 significantly reduced serum phosphate, but not serum calcium, on day 7 after injection (Fig. 1A). Agonistic activity of R1MAb1 requires the presence of both of the two Fab arms in the molecule; an engineered one armed version of R1MAb1 (OA-R1MAb1) with only one Fab arm binds to FGFR1, but its agonistic activity is largely compromised [22]. When injected into diabetic *db/db* mice at 3 mg/kg, R1MAb1, but not OA-R1MAb1, reduced serum phosphate levels, suggesting that agonistic activity is required for the phosphate reducing activity (Fig. 1B). Neither molecule affected serum calcium levels (Fig. 1B). To investigate whether the phosphate reducing activity is an on-target effect through activation of FGFR1, we injected another agonistic anti-FGFR1 antibody, R1MAb2, into lean C57BL/6 mice at 1 mg/kg. Serum and tissue were collected 48 hours post injection. Since R1MAbs decrease food intake and body weight when injected into mice [22], control mice injected with an isotype control IgG were subjected to pair-feeding to adjust the food intake and body weight (Fig. 1C). The serum phosphate levels were significantly lower in R1MAb2 injected mice compared to pair-fed control mice, indicating that the decrease in serum phosphate levels were indeed the result of FGFR1 activation (Fig. 1A). Again, no change in serum calcium was observed (Fig. 1C).

**Figure 2:**
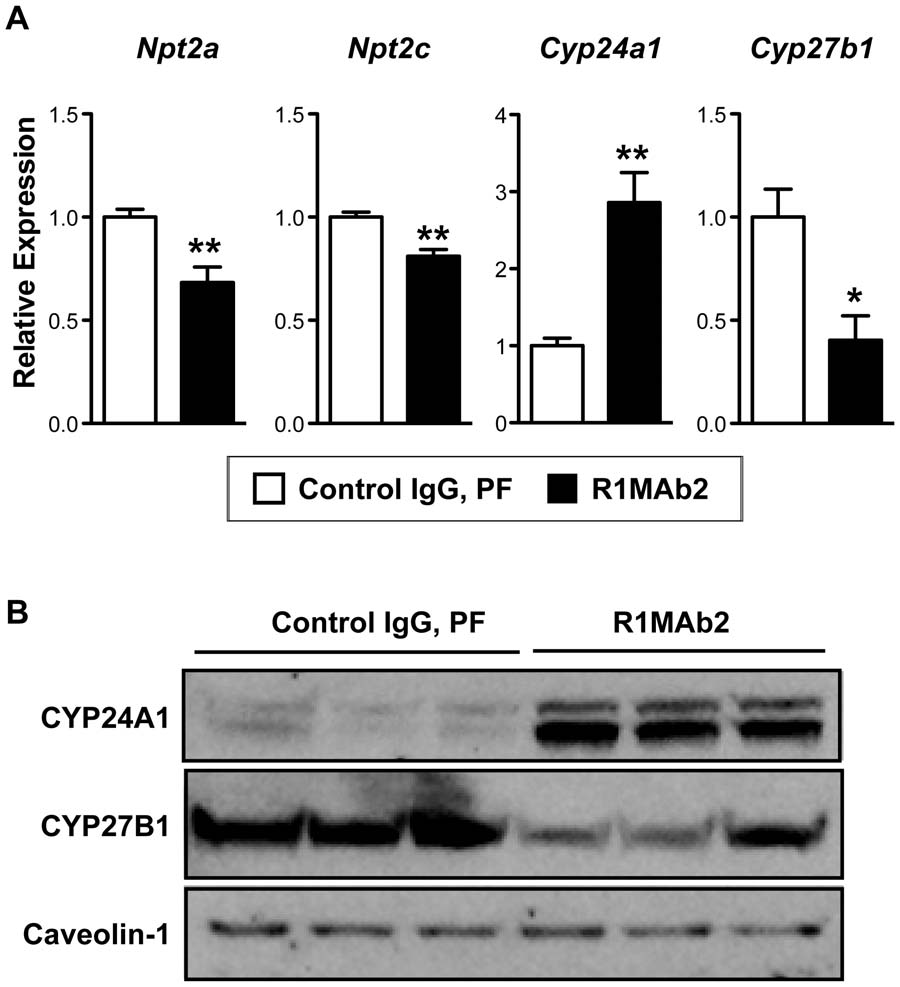
R1MAb2 activates the FGF23 pathway in kidney cortex. (**A**) mRNA expression analysis by qPCR in kidney cortex at 48 hour post injection. The same animals in Figure 1C were analyzed. N = 8 mice/group. * p<0.01, ** p<0.005. (**B**) Protein expression by western blot on the membrane fraction of kidney cortex homogenate at 48 hour post injection. Each lane represents an independent sample from different animals. N = 3 mice/group.

Serum phosphate levels are regulated in the kidney by the action of phosphaturetic hormone FGF23. In the kidney epithelial cells, FGF23 regulates expression of multiple genes involved in phosphate secretion and vitamin D metabolism by activating multiple FGFRs including FGFR1, FGFR3, and FGFR4 [4], [6], [23]. To determine whether selective activation of FGFR1 by R1MAb leads to induction of the FGF23 program, we determined expression of FGF23 target genes in the kidney cortex in mice treated with R1MAb2 by real-time quantitative PCR (qPCR). Consistent with the idea that the FGF23 program is activated in the kidney in R1MAb2-treated mice, R1MAb2 suppressed expression of *Npt2a*, *Npt2c*, and *Cyp27b1* mRNAs and induced expression of *Cyp24a1* mRNA (Fig. 2A). Similar changes in CYP24A1 and CYP27B1 proteins were also observed by western blotting (Fig. 2B).

### R1MAb induces FGF23 secretion

**Figure 3:**
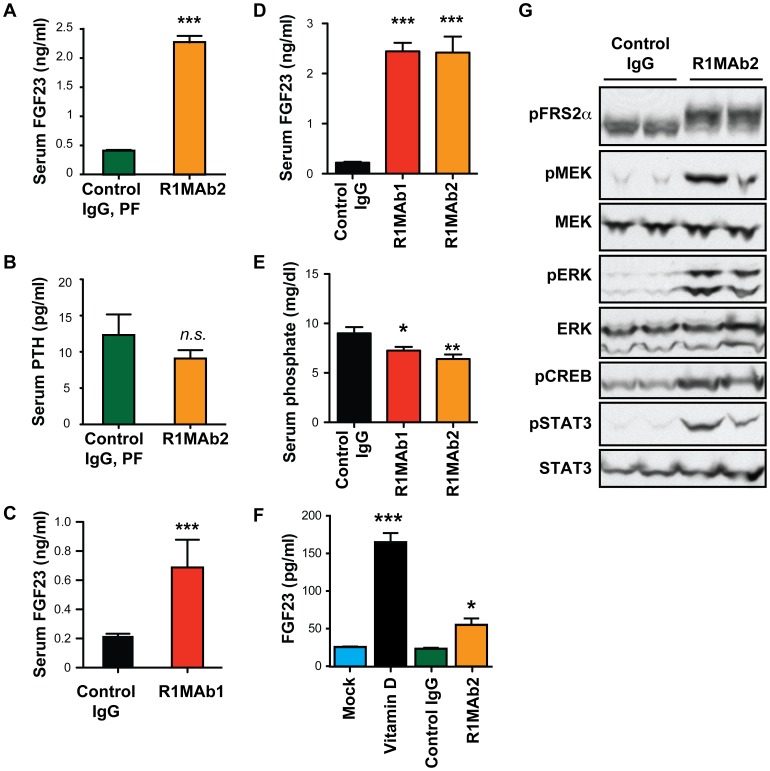
R1MAb2 induces FGF23 production. (**A and B**) Serum FGF23 (A) and PTH (B) levels in male C57BL/6 mice intraperioneally injected with R1MAb2 or isotype control (Control IgG) at 1 mg/kg. The same animals described in Figure 1C and Figure 2 were analyzed at 48 hour post injection. N = 8 mice/group. (**C**) Serum FGF23 levels in female *db/db* mice intraperioneally injected with R1MAb1 or isotype control (Control IgG) at 2 mg/kg. The samples were collected at 7 days post injection. N = 6 mice/group. (**D and E**) Serum FGF23 levels (D) and phosphate levels (E) in male C57BL/6 mice intraperioneally injected with an indicated antibody at 1 mg/kg. The samples were collected at 3 days post injection. N = 8 mice/group. (**F**) FGF23 levels in culture medium after treatment of differentiate rat osteoblast with vitamin D (100 nM), R1MAb1, or isotype control IgG (26.7 nM). The cells were incubated for 48 hours in the presence of the indicated ligand. N = 6 samples/treatment. (A–F) * p<0.05, **<p<0.005, ***<p<0.0005. (**G**) Differentiated rat osteoblasts were treated with R1MAb2, or isotype control IgG (26.7 nM), for 1 hour and subjected to Western blot analysis to examine phosphorylation of MAPK pathway proteins, CREB and STAT3.

To test whether the observed dysregulation of FGF23-target genes in the kidney by R1MAb2 can be attributed to an increase in circulating FGF23, we determined serum FGF23 levels in R1MAb2-treated C57BL/6 mice by specific ELISA. This revealed a five-fold elevation in serum FGF23 (Fig. 3A), and no change in serum parathyroid hormone (PTH) level (Fig. 3B). Thus, R1MAb2 increases circulating FGF23 protein levels to reduce serum phosphate levels independently of PTH. An increase in serum FGF23 was also observed in diabetic *db/db* mice treated with R1MAb1 (Fig. 3C). A side-by-side comparison of R1MAb1 and R1MAb2 in C57BL/6 mice indicated that both antibodies induce similar effects on serum FGF23 and phosphate levels (Fig. 3D and E).

Skeletal production of FGF23 is regulated by multiple hormonal factors, including PTH, vitamin D and serum phosphate to maintain phosphorus homeostasis [24], [25]. To investigate whether R1MAb acts directly on bone cells to induce FGF23 production, we treated differentiated primary rat calvariae osteoblast cells with R1MAb2 and examined FGF23 protein secretion into medium. In this cell type, vitamin D induced FGF23 production as previously reported (Fig. 3F) [24]. We also found that R1MAb2 increased production of FGF23 protein after a 48-hour treatment (Fig. 3F). The activity of R1MAb2 is likely mediated by activation of FGFR1 and downstream mitogen-activated protein kinase (MAPK) signaling cascades as R1MAb2 induced phosphorylation of MAPK signaling intermediates (Fig. 3G). R1MAb2 also increased the levels of phosphorylated cAMP response element-binding protein (CREB) and phosphorylated Signal transducer and activator of transcription 3 (STAT3), two downstream transcription factors known to mediate FGFR signaling (Fig. 3G).

**Figure 4:**
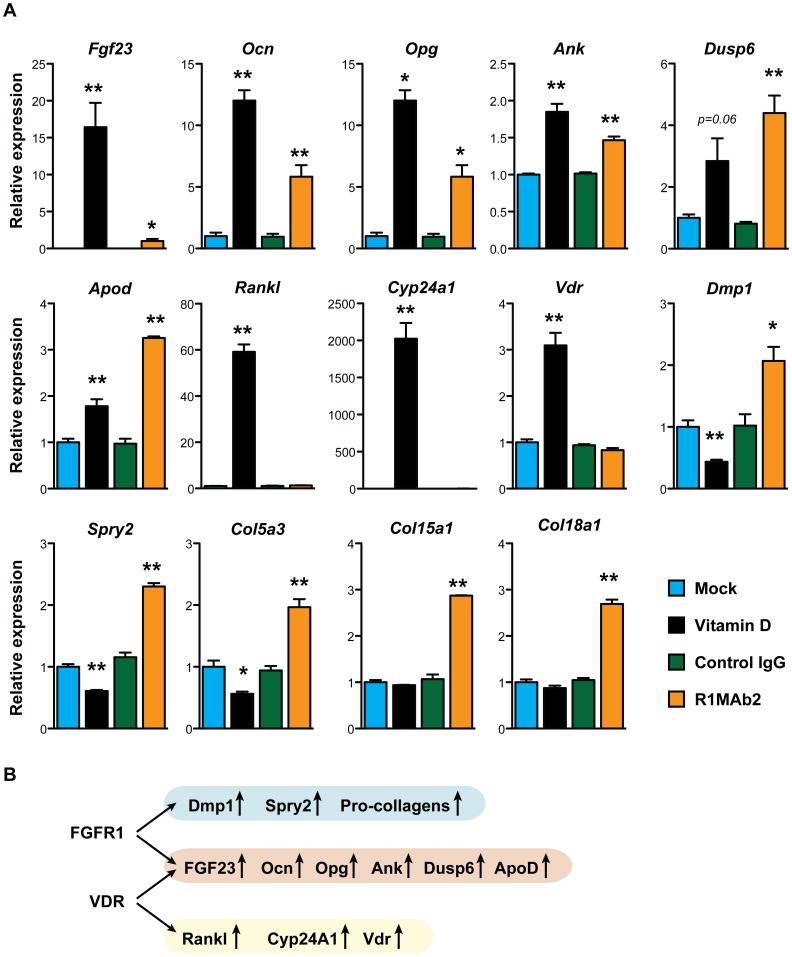
Gene expression in differentiated osteoblasts. (**A**) mRNA was isolated from differentiated osteoblasts treated with the indicated ligands (vitamin D (100 nM), R1MAb2, or isotype control IgG (26.7 nM)) for 48 hours, and subjected to qPCR analysis. Data represent means ± SEM (N = 3). *P<0.05, **P<0.01, versus mock (for vitamin D) or versus control IgG (for R1MAb2). (**B**) Schematic summary of the data presented in (A). Vitamin D and R1MAb2 induce overlapping, but distinct sets of target genes.

To determine whether R1MAb2 induces expression of *Fgf23* mRNA, we carried out mRNA expression analysis by qPCR. As shown in Figure 4A, both vitamin D and R1MAb2 increased *Fgf23* mRNA, indicating that regulation occurs at the transcriptional level in both cases. In addition to *Fgf23*, R1MAb2 and vitamin D treatment similarly induced the expression of several genes, including *osteocalcin* (*Ocn*), *osteoprotegerin* (*Opg*), *progressive ankylosis* (*Ank*), *dual-specificity phosphatase 6* (*Dusp6*), and *apolipoprotein D* (*Apod*). However, we also found genes that were regulated by only vitamin D, but not by R1MAb2 (*rank ligand* (*Rankl*), *Cyp24a1*, and *vitamin D receptor* (*Vdr*)). We also found genes that were induced by only R1MAb2, but not by vitamin D (*dentin matrix acidic phosphoprotein 1* (*Dmp1*), *Sprouty 2* (*Spry2*), and *pro-collagens* (*Col5a3*, *Col15a1*, and *Col18a1*)) (Fig. 4B). Thus, vitamin D and R1MAb2 act separately, presumably through different transcription factors, but eventually converge on the regulatory region of several commonly regulated genes including the *Fgf23* gene.

### The effect of FGFR1 activation on bone density

**Figure 5:**
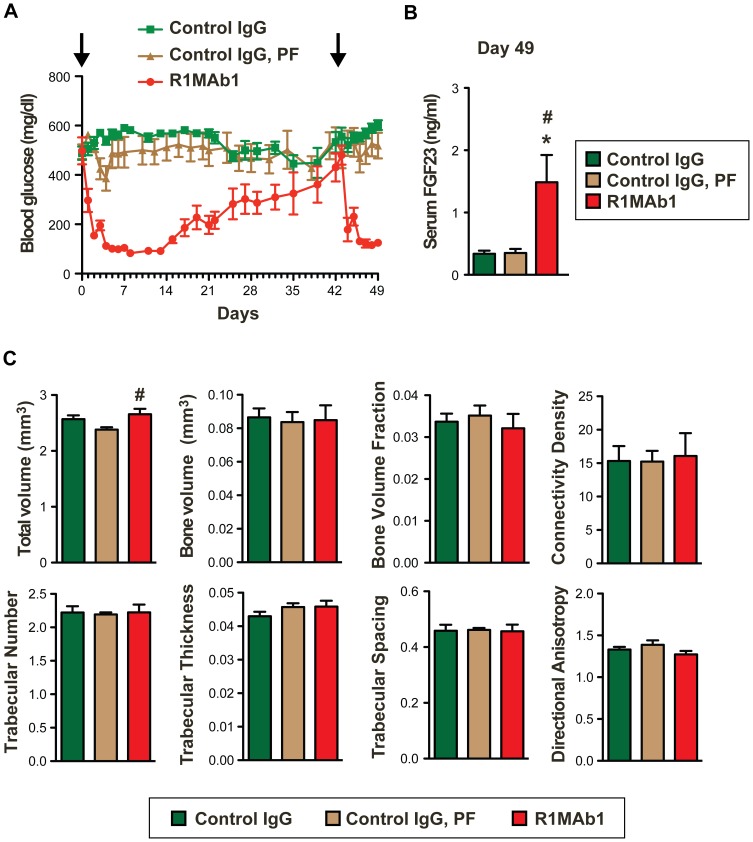
Bone effects of R1MAb2 treatment. (**A**) Blood glucose levels of female *db/db* mice (N = 8 mice/group) during the study. The mice received intraperitoneal injection of R1MAb1 or control IgG at 3 mg/kg doses on day 0 and day 42 (Arrow). Statistical significance in glucose reduction (p<0.05) was observed between day 3–31 and day 43–49. (**B**) Serum FGF23 levels of mice in (A) on day 49. p<0.01, N = 7–8 mice/group. (*# p<0.05, versus Control IgG (*) or versus Control IgG, PF (#)) (**C**) Bone phenotype of mice described in (A–B). The bones were dissected on day 49, and subjected to μCT analysis. Statistical significance (p<0.05) was observed only for total volume, but not other parameters shown. # p<0.05 (versus Control IgG, PF).

FGF23 hyperactivity and the resulting hypophosphatemia are associated with reduced bone density in tumor-induced osteomalacia and hereditary hypophosphatemic rickets [4]–[7]. Therefore, it is possible that R1MAb decreases bone mineral density by activating FGF23 pathway in the kidney. However, R1MAb also induces expression of *Dmp1* and *Opg* genes that have been implicated in increasing bone mineralization (Fig. 4) [26], [27]. Since anti-FGFR1 antibodies have been implicated for anti-diabetic therapies [22], we investigated the effects of R1MAb on bone density in diabetic *db/db* mice. The use of *db/db* mice allowed us to readily monitor blood glucose level as an indicator of R1MAb activity. Mice were injected with R1MAb1 or isotype control IgG, on two different days of the study: day 0 and 42. Since R1MAb1 reduces food intake [22], one group of control mice were pair-fed (PF) to adjust food intake until day 24. At day 24, the food intake of R1MAb1-treated mice returned to normal, and thus all the mice were fed *ad libitum* after day 24. As we previously demonstrated, R1MAb1 injections achieved sustained glucose lowering (Fig. 5A). Blood glucose was maintained at statistically lower levels by R1MAb1 injections between days 1–32, and again between days 43–49 (Fig. 5A). At euthanasia on day 49, an increase in serum FGF23 level was confirmed as expected (Fig. 5B). The body weight of the three groups was not significantly different at euthanasia (Control IgG: 37.3±1.0 g, Control IgG, PF: 40.2±1.3 g, R1MAb1: 39.6±2.6 g). To quantify trabecular and cortical structure of the mouse femur, micro-computed tomography (μCT) was employed. The trabecular structural characteristics were quantified by direct 3D morphometric analysis (Fig. 5C). To our surprise, R1MAb1 treatment did not affect any of the parameters that we examined, except total bone volume. Total bone volume was slightly, but nonetheless increased in a statistically significantly manner in R1MAb1-treated mice compared with pair-fed mice treated with control IgG. Thus short-term treatment with R1MAb1 and the resulting mild increase in circulating FGF23 were not sufficient to significantly reduce bone mineral density at the dose that was sufficient to reduce blood glucose levels in diabetic *db/db* mice.

### R1MAb acts as an FGF23 mimetic

**Figure 6:**
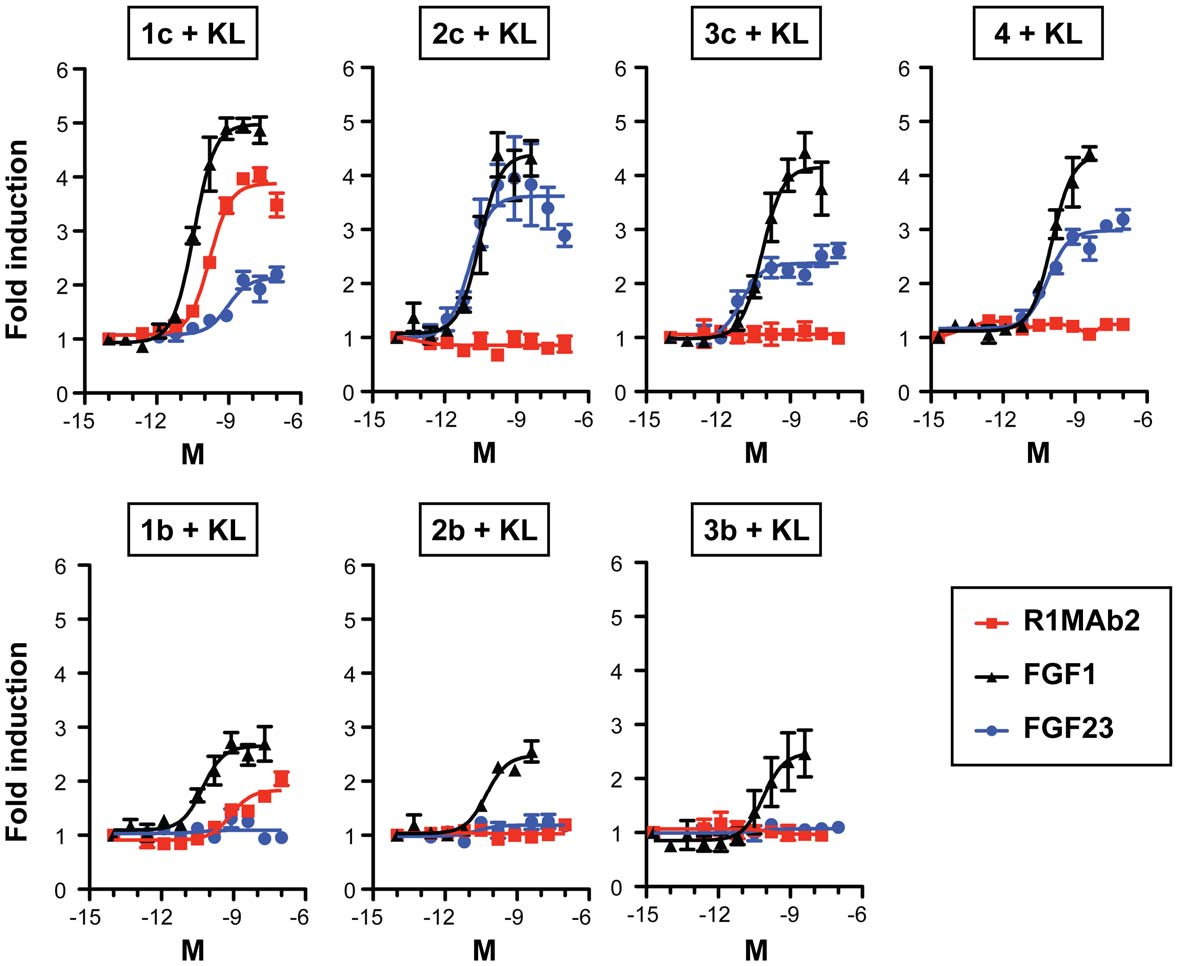
R1MAb2 activates FGFR1 in the presence of Klotho. The activation of FGFR was assessed using GAL-Elk1 luciferase assay in rat L6 myoblast cells. Cells were co-transfected with an expression vector for Klotho (KL) and the indicated FGFR isoform together with GAL-Elk1, SV40-renilla Luciferase, and GAL-responsive firefly luciferase reporter. Transfected cells were incubated with increasing concentrations of R1MAb2, human FGF23, or a positive control, FGF1, for 8 h before luciferase assays. Firefly luciferase activity was normalized to renilla luciferase activity and expressed as fold induction over untreated control. The data represents means ± SEM (N = 3).

FGFR1 is expressed in the kidney epithelium and has been implicated to function as a receptor for FGF23 [13], [17]. Thus, in addition to affecting FGF23 production in bones, R1MAb could activate the FGF23 signaling pathway by directly stimulating the FGFR1/Klotho complex in the kidney. In order to confirm that R1MAb2 can selectively activate FGFR1, but not other FGFRs, even in the presence of Klotho protein, we used the Gal-Elk1-based luciferase assay in rat L6 cells lacking endogenous FGFRs and Klotho, but transfected to express these receptors. As shown in Figure 6, R1MAb2 induced luciferase activity when FGFR1 is expressed in the cells that also express Klotho protein. In this assay system, FGF23 induced luciferase activity when cells expressed Klotho together with FGFR1c, 2c, 3c, or FGFR4, but not with b isoforms of FGFRs (Fig. 6). Therefore, R1MAb2 indeed acts as a FGFR1 selective FGF23 mimetic in this context.

**Figure 7:**
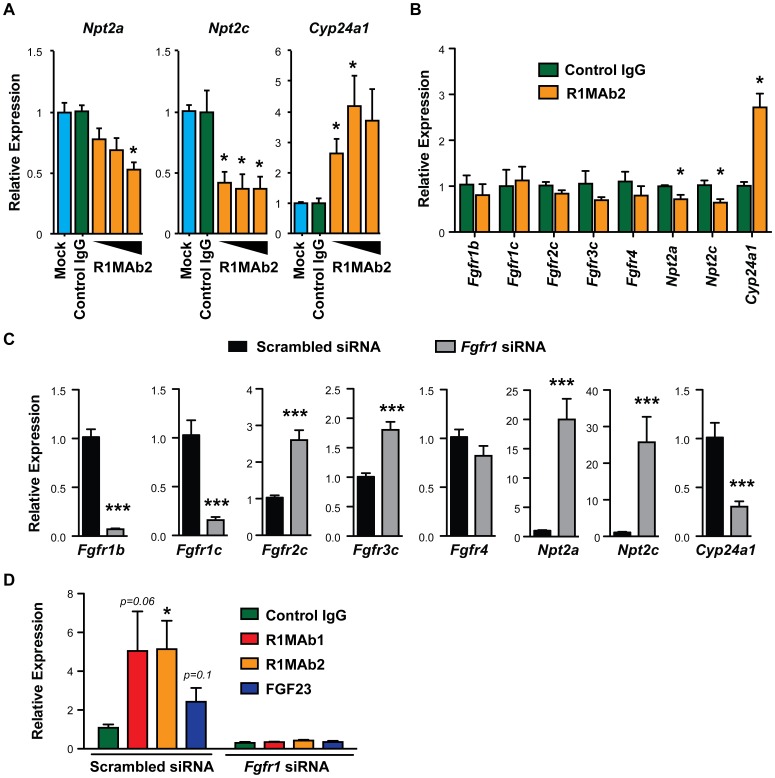
R1MAb2 activates the FGF23 pathway in kidney epithelial cells. (**A**) OK cells were treated with vehicle (Mock), R1MAb2 (0.5, 5, or 50 nM), or isotype control IgG (50 nM) for 24 hours, and the mRNA expression of indicated genes were determined by qPCR. The expression of each gene was normalized by the expression of *actin* in the same sample and shown as relative expression. N = 3. (**B**) Similar gene expression analysis in OK cells after treatment with an indicated antibody at 50 nM. N = 6. (**C**) mRNA expression in OK cells treated with scrambled or FGFR1 siRNA oligos, determined by qPCR. N = 6. (**D**) *Cyp24a1* gene expression in OK cells after treatment with siRNA oligos and an indicated ligand. N = 6. The data represents means ± SEM. * p<0.05 or *** p<0.001 compared with the control group.

To test whether R1MAb2 has the ability to activate FGF23 signaling in kidney epithelial cells, we treated the cultured renal proximal epithelial Opossum Kidney (OK) cells with R1MAb2 and examined expression of known FGF23 target genes [4], [23]. The OK cell line has been shown to respond to recombinant FGF23 to reduce expression of *Npt2a* and reduce phosphate uptake [23], [28]. Consistent with the idea that activation of FGFR1, but not other FGFRs, is sufficient to induce the signaling pathway downstream of FGF23, R1MAb2 treatment reduced mRNA expression of *Npt2a* and *Npt2c*, and induced expression of *Cyp24a1* in cultured OK cells (Fig. 7A and B). Treatment of the OK cells with R1MAb2 by itself did not change the level of expression of *Fgfrs* significantly (Fig. 7B). Although OK cells respond to FGF23, we failed to detect *Klotho* mRNA by qPCR, suggesting that *Klotho* mRNA is expressed at extremely low levels, if at all.

**Figure 8:**
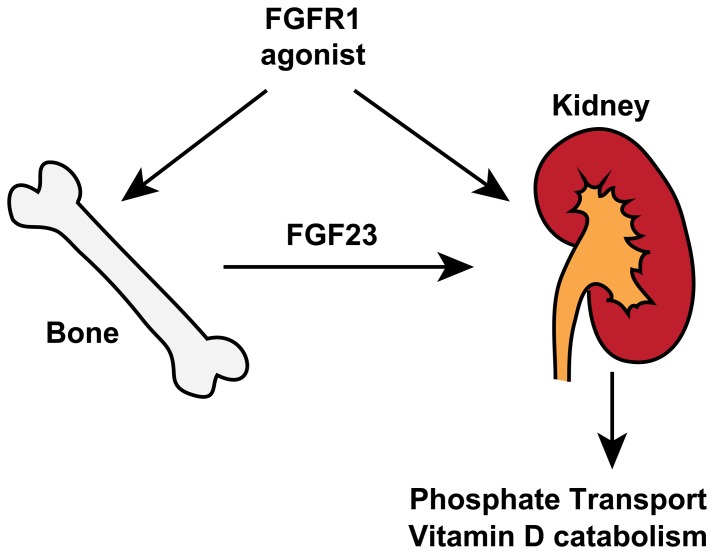
A model for the mechanism of perturbation of phosphate homeostasis by anti-FGFR1 agonists. See text for explanations.

To examine the contribution of FGFR1 to the expression of genes in the FGF23 pathway, we designed small interfering RNAs (siRNA) against *Fgfr1* in OK cells (Fig. 7C). While our *Fgfr1-specific* siRNA oligos knocked down *Fgfr1* mRNA expression efficiently, *Fgfr2c*, and *3c* were significantly upregulated in the siRNA-treated cells, suggesting receptor compensation in the absence of FGFR1. Strikingly, knockdown of *Fgfr1* led to over a 20-fold induction of *Npt2a* and *Npt2c* and a significant reduction in *Cyp24a1* expression. These findings correspond with previous data showing that ligand-based activation of the FGFR1 regulates the expression of these genes (Fig. 7A and B). This also suggests that the basal activity of FGFR1 in the absence of ligand addition is sufficient to regulate these downstream genes. Furthermore, knockdown of *Fgfr1* blunted the *Cyp24a1* response upon R1MAb1, R1MAb2, and FGF23 treatment (Fig. 7D). Taking all the results together, we propose that R1MAbs act by both activating FGF23 receptor complex in the kidney epithelium, as well as affecting the production of FGF23 directly in bone (Fig. 8).

## Discussion

A number of previous studies have implicated FGF23 and FGFRs in the regulation of phosphate metabolism; however, the identity of FGFR isoforms that are functionally important in phosphate regulation remains elusive. In the current study, we used selective antibody activators of FGFR1 to study the effects of systemic FGFR1 activation on phosphate homeostasis. Our results demonstrate that activation of FGFR1, but not other FGFRs, is sufficient to induce FGF23 expression and the resulting hypophosphatemia in adult mice. In addition, FGFR1 activation in cultured kidney epithelial cells is sufficient to induce FGF23 program. siRNA-mediated *Fgfr1* knockdown induced the opposite effects. These results complement previous genetic studies with mice deficient for each FGFR isoform, and suggest the central role of FGFR1 in the FGF23 signaling network (Fig. 8).

Antibody-mediated activation of FGFR1 is potentially mimicking some aspect of osteoglophonic dysplasia (OGD), a rare genetic disorder characterized by a distinctive skeletal dysplasia caused by an activating FGFR1 germ line mutation [21]. FGFR1 plays important roles during embryonic development, in particular in skeletal development [29]. Individuals with OGD thus display mild to severe skeletal malformation including craniosynostosis and dwarfism [21]. In addition, they also exhibit hypophosphatemia, which is likely to be independent of craniosynostosis, dwarfism, or other developmental problems. The mechanistic basis for hypophosphatemia in OGD is unclear, although one individual with hypophosphatemia has been found to have elevated circulating FGF23 levels [21]. Together with our results, it is conceivable that genetic activation of FGFR1 in bones leads to elevated FGF23 in OGD, resulting in hypophosphatemia.

Typical OGD individuals also exhibit osteomalacia, which might be secondary to hypophosphatemia. Continuous production of recombinant FGF23 from implanted cell line was also shown to induce marked hypophosphatemia (∼50% of normal) and osteomalacia in nude mice at day 45 after the implantation [5]. Intriguingly, pharmacological activation of FGFR1 by R1MAb1 injection did not result in an apparent defect in bone mineral density in *db/db* mice despite the ability of R1MAb1 to induce FGF23 production and hypophosphatemia. The method used for this study cannot directly assess the non-calcified osteoid volume, thus it is formally possible that R1MAbs affect osteoid formation independently of calcified bone volume in this timeframe. Also the lack of bone effect could be simply due to rather mild effects of R1MAb1 on phosphate regulation and short exposure. Further analysis is required to determine whether more frequent and long-term administration with R1MAb (or other FGFR1 activating agents) could significantly affect bone mineralization or relative osteoid volume.

In addition to FGF23, our gene expression studies in cultured differentiated osteoblasts identified a number of R1MAb target genes, which could also be altered in OGD patients. Many of these genes have been implicated in regulation of bone mineral density or function. For example, R1MAb2 induced *Opg*, a gene encoding a secreted protein that inhibits RANKL and osteoclast differentiation [27], [30]. Dmp1 deficiency is implicated in hypophosphatemia and osteomalacia in humans [31], while overexpression promotes bone mineralization [26]. In our assays, we found that R1MAb2 treatment upregulated both *Opg* and *Dmp1*. Induction of these genes by FGFR1 activation *in vivo* should promote bone mineralization. We also found that R1MAb2 treatment upregulated *Ank*, whose induction *in vivo* may promote bone loss. *Ank* encodes multipass transmembrane protein that functions in pyrophosphate regulation [32]. Loss of *Ank* in humans leads to craniometaphyseal dysplasia characterized by progressive thickening of bones [33]. As expected, R1MAb also induced negative feedback regulators of FGFR signaling, *Spry2* and *Dusp6* [34]. Thus, FGFR1 activation could perturb bone mineralization through multiple mechanisms.

Pharmacological modulation of FGFR1 activity has been explored as therapeutic strategy in several diseases. For example, FGFR1 overexpression or a somatic activating mutation in the kinase domain has been found in tumor cells, leading to the examination of FGFR1 as an anti-cancer target [34], [35]. Modulation of FGFR pathway has been implicated in major mood depressive and other psychiatric disorders [36], [37]. In addition, we recently provided evidence of FGFR1 activation in adipose tissues as a therapeutic target for the treatment of insulin resistance and type 2 diabetes [22]. Not surprisingly, one common side effect of characterized FGFR modulators is an alteration in phosphate homeostasis. For example, pan-FGFR inhibitors PD173074 and PD176067 both induced hyperphosphatemia and changes in other FGF23-related parameters [38], [39]. Interestingly, both inhibitory PD173074 and the activating anti-FGFR1 antibodies described here increase serum FGF23 levels. PD173074 is proposed to act primarily in the kidney to increase serum vitamin D and phosphate, which in turn induces FGF23 production in bone through an FGFR-independent mechanism [39]. We believe that R1MAbs function directly in the bone to induce FGF23 secretion. Thus, FGFR1 activation is sufficient, but not necessary, for skeletal production of FGF23 *in vivo*.

In conclusion, the work presented here demonstrates that pharmacological FGFR1 activation is sufficient to increase FGF23 production and decrease serum phosphate levels in adult mice. These findings provide new insights into the mechanistic basis for human hypophosphatemic disorders such as OGD and hypophosphatemic rickets, associated with an increase in circulating FGF23 and the resulting urinal phosphate wasting. FGFR1 activation could affect phosphate homeostasis at least in two tissues: bone and kidney (Fig. 8). Thus, tissue specific modulation of FGFR1 is likely necessary for therapeutic intervention for metabolic diseases or psychiatric disorders, to gain efficacy without adverse side effects related to the FGF23-axis. In addition, tissue specific modulation of FGFR1 can conceivably counteract conditions associated with FGF23 resistance or chronic hyperphosphatemia, such as those seen in patients with chronic kidney disease [40]. Further studies are warranted to elucidate the physiological pathways downstream of FGFR1 or other FGFRs.

## Materials and Methods

### Research ethics

All animal studies were conducted in accordance with the Guide for the Care and Use of Laboratory Animals, published by the National Institutes of Health (NIH) (NIH Publication 8523, revised 1985). The Institutional Animal Care and Use Committee (IACUC) at Genentech reviewed and approved all animal protocols. The approval IDs for this study are: #09-2564, #10-0793, #10-1299D, #11-0277, #11-0909, #12-2555B.

### Mouse studies

Mice were maintained in a pathogen-free animal facility at 21°C under standard 12 hr light/12 hr dark cycle with access to chow (a standard rodent chow, Labdiet 5010, 12.7% calories from fat) and water ad libitum. Male C57BL/6 mice or female *db/db* mice in a C57BLKS/J background were purchased from Jackson Laboratory. For high-fat diet feeding, a high fat, high carbohydrate diet (Harlan Teklad TD.03584, 58.4% calories from fat) was used. Serum inorganic phosphate and calcium levels were determined by Cobas Integra 400 Chemistry Analyzer (Roche). Serum FGF23 levels were determined by ELISA (Immutopics). Blood glucose levels were determined using a Contour glucose meter (Bayer).

### Ligands

Recombinant anti-FGFR1 antibodies and isotype controls (Herceptin or anti-Ragweed) were produced in CHO cells and purified to homogeneity in PBS. Production of OA-R1MAb1 was previously described [22]. Ligands used were Human FGF1 (Peprotech), human FGF23 (R&D systems), and vitamin D (Calcitriol/1,25-dihydroxyvitamin D_3_) (Sigma-Aldrich).

### Cell culture

All the cells were maintained at 37°C under 5% CO_2_. OK and L6 cells were purchased from American Tissue Type Culture Collection (ATCC) and cultured in Dulbecco's modified eagle medium (DMEM) containing 4.5% glucose, L-glutamine, 10% fetal bovine serum (FBS) and penicillin-streptomycin. Rat primary calvariae osteoblast cells were purchased from Lonza and differentiated for 21 days according to manufacturer's instruction. For gene expression analysis in OK cells, the cells were plated at a density of 1.2×10^5^ cells/well in six-well plates and cultured for 24 hours in culture media containing 10% FBS. The cells were serum starved for 24 hours by changing to the serum-free medium containing 0.2% BSA, and then treated with appropriate recombinant proteins for 24 hours. siRNA oligos for opossum (*Monodelphis domestica*) *Fgfr1* were designed using Dharmacon's siRNA design program. Two siRNA oligos (GATGGAAGTGCTACATTTATT and GAGGTGAATGGAAGTAAGA) were independently validated and used as a pool at a working concentration of 30 nM each for the experiments described. Oligos were transfected into cells plated in a six-well plate at a density of 1.6×10^5^ cells/well using Lipofectamine-RNAiMax (Life Technologies). Following 48-hour incubation post transfection, cells were serum starved for 24 hours. Cells were then treated with an indicated ligand for an additional 24 hours.

### Luciferase assay

Rat L6 myoblasts in a 96-well plate were transiently-transfected with expression vectors encoding Renilla luciferase (pRL-SV40, Promega), human Klotho, appropriate human FGFR, GAL4-Elk-1 transcriptional activator (pFA2-Elk1, Stratagene), and firefly luciferase reporter driven GAL4 binding sites (pFR-luc, Stratagene), using FuGENE HD Transfection Reagent (Promega). On the next day, the transfected cells were cultured for an additional 8 hours in serum free media containing 25 µg/ml porcine heparin (Sigma) and appropriate recombinant protein at various concentrations. The cells were then lysed with PLB reagent (Promega) and luciferase activity in each well was determined using Dual-Glo Luciferase Assay System (Promega) and EnVision Multilabel Reader (Perkin Elmer). Firefly luciferase activity was normalized to the co-expressed Renilla luciferase activity.

### Gene expression analysis

To isolate RNA from mouse kidney cortex, the whole kidney was dissected from the animal and immediately soaked in RNAlater RNA stabilization solution (Qiagen). The kidney cortex was dissected and total RNA was isolated using QIAzol reagent (Qiagen), and then further purified using RNeasy kit. For gene expression analysis in OK cells and rat osteoblasts, total RNA was isolated with RNeasy kit (Qiagen). Total RNA was used to synthesize cDNA using the Quantitect Reverse Transcription Kit (Qiagen) or High-Capacity cDNA Reverse Transcription Kit (ABI, Foster City, CA). For qPCR, samples were run in triplicate in the ABI Prism 7900HT (Applied Biosystems) by using SYBR green universal mix (Invitrogen) or by Taqman universal mix (Roche) and normalized by levels of 36B4 or actin as indicated. Pre-designed Quantitect primers from Qiagen or in-house designed primers designed using primer express software (Applied Biosystems) were used. Sequences of in-house designed primers will be provided upon request.

### Western blot

To isolate the kidney membrane fraction, kidney cortex was dissected and homogenized in homogenize buffer (50 mM Tri-HCl (pH7.5), 1 mM EDTA, 0.3 M Sucrose, proteinase inhibitor cocktail (Roche) on ice. The homogenates were centrifuged 3,000×g at 4°C for 5 min. The supernatant were collected and further centrifuged at 300,000×g for 30 min at 4°C. The pellet was resuspended in 200 µl RIPA buffer containing proteinase inhibitor cocktail, and then centrifuged at 20,000×g for 10 min. The supernatant was collected and 50 µg protein was loaded onto each well for SDS PAGE. Cell extracts from differentiated rat osteoblasts were generated by lysing cells in 2× LDS buffer (Invitrogen) containing protease and phosphatase inhibitor tablets (Roche). Samples were then sonicated and used for Western blot analysis by standard methods. Antibodies used for Western blot analysis were from Cell Signaling Technology: pFRS2α (Y196) (#3864), pMEK1/2 (S217/221) (#9154), pERK1/2 (T202/Y204)(#4370), pStat3 (S727) (#9134), pCREB (S133) (#9198), ERK1/2 (#4695), Stat3 (#9132), MEK (#9126).

### μCT analysis

Femur samples were imaged by a SCANCO Medical (Basserdorf, Switzerland) μCT40 micro-imaging system operating with x-ray tube energy level a 70 keV and a current of 114 microamperes. Contiguous axial image slices were obtained with an isotropic voxel size of 12 µm. Morphometric analysis of the trabecular bone within the femur was performed with the SCANCO Medical (Basserdorf, Switzerland) μCT40 evaluation software. Semi-automated contouring was used to define a volume of interest (VOI), comprising secondary trabecular bone dorsal to the proximal femur growth plate and extending 1.5 mm distal to primary trabecular bone. The cortical bone was excluded by placement of the VOI boundaries within the inner boundary of the cortical bone. Prior to image segmentation, a constrained three-dimensional (3D) Gaussian low-pass filter was applied to the image data for noise suppression (filter sigma = 0.5, filter support = 1). A global threshold (0.36 gHA/cm3) was applied to extract a “binarized” trabecular structure from the VOI. The trabecular segmentation threshold was chosen by visual inspection of the segmentation results from a representative subset of the samples. The trabecular structural characteristics were quantified by direct 3D morphometric analysis [41]. Previous studies have shown that morphometric analysis of trabecular bone by micro-computed tomography is well correlated with similar estimates made by histomorphometry [42].

### Statistics

For the results presented in Figure 5, one-way ANOVA with post-hoc Dunnett's test was used for statistical analysis to compare treatment groups. For other results, unpaired student's t-test (two-tailed) was used. A p-value<0.05 was considered statistically significant. Values were presented as means ± SEM.
